# Setup of Extruded Cementitious Hollow Tubes as Containing/Releasing Devices in Self-Healing Systems

**DOI:** 10.3390/ma8041897

**Published:** 2015-04-21

**Authors:** Alessandra Formia, Salvatore Terranova, Paola Antonaci, Nicola Maria Pugno, Jean Marc Tulliani

**Affiliations:** 1 Department of Applied Science and Technology, INSTM Reference Laboratory for Ceramics Engineering, Politecnico di Torino, Corso Duca degli Abruzzi 24, I-10129 Torino, Italy; E-Mails: alessandra.formia@polito.it (A.F.); slv.terranova@gmail.com (S.T.); 2 Department of Structural, Geotechnical and Building Engineering, Laboratory of Bio-Inspired Nano-Mechanics “Giuseppe M. Pugno”, Politecnico di Torino, Corso Duca degli Abruzzi 24, I-10129 Torino, Italy; E-Mail: paola.antonaci@polito.it; 3 Laboratory of Bio-Inspired & Graphene Nanomechanics, Department of Civil, Environmental and Mechanical Engineering, University of Trento, Via Mesiano, 77, I-38123 Trento, Italy; E-Mail: nicola.pugno@unitn.it; 4 Center for Materials and Microsystems, Fondazione Bruno Kessler, Via Sommarive 18, I-38123 Povo (Trento), Italy; 5 School of Engineering & Materials Science, Queen Mary University of London, Mile End Road, London E1 4NS, UK

**Keywords:** concrete, microcracking, mechanical properties, durability, self-healing

## Abstract

The aim of this research is to produce self-healing cementitious composites based on the use of cylindrical capsules containing a repairing agent. Cementitious hollow tubes (CHT) having two different internal diameters (of 2 mm and 7.5 mm) were produced by extrusion and used as containers and releasing devices for cement paste/mortar healing agents. Based on the results of preliminary mechanical tests, sodium silicate was selected as the healing agent. The morphological features of several mix designs used to manufacture the extruded hollow tubes, as well as the coatings applied to increase the durability of both core and shell materials are discussed. Three-point bending tests were performed on samples produced with the addition of the above-mentioned cementitious hollow tubes to verify the self-healing effectiveness of the proposed solution. Promising results were achieved, in particular when tubes with a bigger diameter were used. In this case, a substantial strength and stiffness recovery was observed, even in specimens presenting large cracks (>1 mm). The method is inexpensive and simple to scale up; however, further research is needed in view of a final optimization.

## 1. Introduction

Concrete is the most widely used construction material because of its high compressive strength and relatively low cost. However, it is very sensitive to crack formation, because of its limited tensile strength. Cracks endanger the durability of concrete structures, as aggressive liquids and gasses may penetrate into the matrix along these cracks and cause further damage in the reinforcement bars [1]. It is for these reasons that a self-healing ability would be strongly desirable for concrete [2], and many efforts have been made by the scientific community in recent years in this direction, as briefly summarized in the following. However, the possible processes of self-healing in cementitious composites using extruded cementitious hollow tubes as containing/releasing devices for healing agents have not been investigated so far, hence comes the idea of the experimental study presented in this paper, which is aimed at providing a contribution to the development of an effective solution for self-healing concretes and mortars.

In the case of aboveground structures, several types of healing agents have been already proposed, mostly single-component, air-curing healing agents, such as cyanoacrylates, epoxy, silicon or alkali-silica solutions, which are preferred to multi-component healing agents, because incomplete mixing of the different components is feared [2]. However, the potentially short shelf life of single-component healing agents might be disadvantageous, as multi-component healing agents have more stability than single-component healing agents, because they are activated later, *i.e*., *in situ*. Therefore, multi-component solutions, such as methyl methacrylate systems and two-component epoxy resins, could be highly desired, too [2]. Polyurethane has also been tested and proved to be a very appropriate healing agent in cementitious materials. The key factor of this healing agent is its ability to expand, so that cracks become sealed, reducing the potential for reinforcing steel corrosion [2].

The self-healing phenomenon of autogenous healing in concretes using geo-materials for practical industrial application has been investigated, too [3], with some encouraging results in terms of small crack sealing and larger crack reduction. In this case, no encapsulation system was used.

Another inorganic healing agent, sodium silicate (Na_2_SiO_3_), was evaluated in [4,5]. It was encapsulated in such a way that it could react with Ca(OH)_2_, as it is naturally present in concrete, to form calcium silicate hydrate products (C–S–H) that heal the crack.

A bio-inspired approach to self-healing concrete has been studied also: bacteria are immobilized in concrete and activated if water permeates into fresh cracks, where they start to precipitate minerals [6,7,8]. The results show that the applied two-component bio-chemical healing agent, consisting of a mixture of bacterial spores and calcium lactate, can be successfully applied to promote and enhance the self-healing capacity of concrete [7,8]. Moreover, as the metabolically-active bacteria consume oxygen, the healing agent may act as an oxygen diffusion barrier, protecting steel rebars against corrosion. However, the long-term (years) durability and cost efficiency of this novel type of concrete need to be resolved before practical applications can be considered [8].

It is obvious that the effectiveness of the self-healing process is not only dependent on crack width, which dictates the capillary forces, but also on the viscosity of the repairing agent: the lower the viscosity, the larger the potential repaired area.

From a mechanical point of view, another prerequisite that should be desirable for the agent is that it could form a sufficiently strong bond between the fracture surfaces in order to prevent the reopening of the crack, thus transferring stresses to other sections and hence increasing the total fracture energy that is required to break the element [1].

The state-of-the-art of self-healing in cementitious materials is well described in [1].

In many investigations, hollow glass tubes were used as encapsulation devices [1]. In that case, the release of the healing agent is activated by crack formation, which results in the breakage of the embedded brittle glass tubes. The internal diameter of the tubes usually ranges from 0.8 mm to 4 mm. However, glass capsules, used in most studies, may have a negative effect on concrete durability, because of the possible onset of alkali-silica reactions. Ceramic capsules have therefore been successfully experimented on [2], in addition to spherical or cylindrical polymeric capsules [1]. In the case of spherical capsules, the capsule diameters reported in the literature range from 5 μm up to 5 mm. For cylindrical capsules, the diameters range from 0.8 mm up to 5 mm [1].

Taking the scientific results summarized above as a starting point, the aim of this research is to produce self-healing cementitious composites based on the use of cylindrical capsules in the shape of small/big extruded cementitious hollow tubes containing sodium silicate as a repairing agent. The proposed system is first described in Section 2, where the experimental details of the hollow tubes’ production are reported, as well as the indication of the methods of analysis adopted to characterize the tubes, the healing agent and a prototype of a self-healing cementitious element. Then, the main results obtained are reported and discussed in Section 3.

A sodium silicate solution (Sigma Aldrich, Na_2_O 10.6 wt%, SiO_2_ 26.5 wt%, H_2_O 62.9 wt%) was selected as a healing agent, considering its low viscosity and good compatibility with cementitious materials, especially when the potential risks of alkali-silica reaction (ASR) could be excluded. As discussed in [5], sodium silicate is able to form some solid phases in the cracks, which allow a recovery of strength and stiffness, after 28 days. In fact, a portion of sodium silicate reacts with the available Ca^2+^ cations of the cement paste, producing calcium silicate hydrates (C–S–H), while the remaining part of the sodium silicate crystallizes after the evaporation or the absorption of the water.

Two different sizes (in terms of cylinder internal diameter) were proposed and tested in this study, in order to ensure that the capillary attractive force of the crack and the gravitational force on the fluid mass are sufficient to overcome the capillary resistive force of the cylindrical capsules and the negative pressure forces caused by the sealed ends [1].

The idea of using a cement paste as a shell material is suggested by its mechanical properties once hardened, which ideally make it sufficiently strong to survive the fresh concrete mixing stage and sufficiently brittle to be broken as soon as a crack is generated in the surrounding hardened concrete matrix, thus allowing the release of the healing agent.

An extrusion technique was adopted to produce the cementitious hollow tubes, due to its easy implementation and flexibility of use in creating different sizes and shapes, in view of the optimization of the adhesion properties. The extrusion process was made easier by the introduction into the cement paste of an acrylic resin (a copolymer of ethyl acrylate and methyl methacrylate (EA/MMA) in water, as specified in Section 2.1.1). It was added, since it is known from the literature that polymer addition to a cement matrix, if exhibiting a plastic behavior, may enhance the deformability of the composite material [9,10], with positive effects also for the final self-healing concrete product. Moreover, waterborne acrylic resins can produce coatings with excellent adhesion to concrete, while offering lower volatile organic compound (VOC) emissions and, thus, possessing low toxicity [11].

A final coating based on a polyester naval resin was applied to the extruded elements, to further increase their sealing ability and shock resistance.

## 2. Experimental Details

### 2.1. Bucatini and Maccheroni as Small and Big Cementitious Hollow Tubes

#### 2.1.1. Extrusion of the Cement

**Figure 1:**
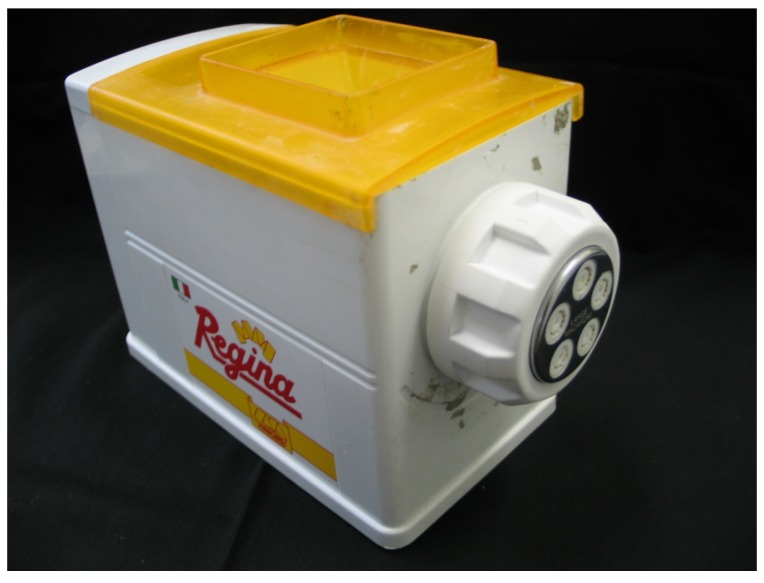
The extruder used for manufacturing *bucatini* and *maccheroni*.

**Figure 2:**
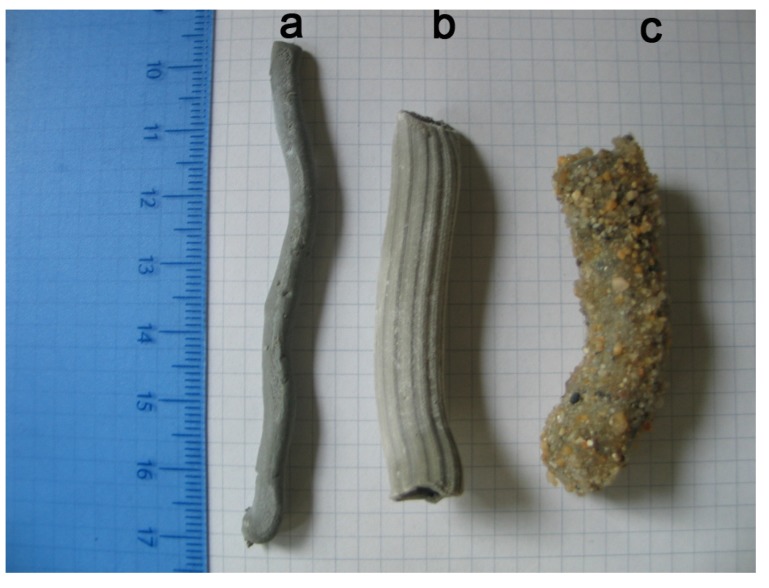
The two typologies of hollow tubes achieved by the extrusion of the cement paste: (**a**) *bucatino* shape; (**b**) *maccherone* shape; (**c**) *maccherone* shape after the application of coatings and sand on the surface.

Two typologies of cementitious hollow tubes with different diameters were obtained by extrusion of a cement paste. A low-cost device normally used to prepare fresh homemade pasta (Regina Wellness, Marcato, Italy) was used to extrude the cement paste. It is composed of a screw extruder having a cylindrical barrel 130 mm long with a diameter equal to 40 mm and two replaceable nozzles (Figure 1). The first one, with a *bucatino* shape, has an external diameter of 5 mm and an internal hole of 2 mm (Figure 2a). The second nozzle has a *maccherone* shape, with a diameter of 10 mm and an internal hole of 7.5 mm (Figure 2b).

The extruder was arranged in such a way that the hollow tubes fall down vertically, to minimize deformation while in the fresh state. Different mix designs were set up in order to define the correct ratio of the components and to achieve objects free from defects, as presented in Section 3.1. Since it is known in the literature that water curing degrades the mechanical strength of polymer-modified cementitious mortars [12], once extruded, the hollow tubes were kept in a moist environment for 7 days (at 20 ± 2 °C and 90% RH) and then in air for other 7 days at room conditions (at 20 ± 2 °C and 50% ± 10% RH) before use.

Concerning the hollow tube mix design, a low amount of water was used to help maintaining the shape during the plastic state of the paste, and several compounds were added to reach a good workability, as reported in the following:

• Portland cement (CEM I 52.5 R, Buzzi Unicem, Italy).
• Copolymer of ethyl acrylate and methyl methacrylate (EA/MMA, Primal B60A, Sinopia, Italy).

The latter was added to reduce the water/cement ratio. This product is an acrylic emulsion of EA/MMA in water (solid content about 50 wt%) and is often used as an additive in lime mortars. Apart from improving chemical resistance, the polymer addition enhances the workability of the paste, allowing the use of a low amount of water [10,12]. The better flow is not only due to the presence of surfactants in the polymers, but is also due to the lower surface tension of polymer molecules, which facilitates better flow of the mix at the same water content [10,12]. Moreover, in the case of the air-cured samples, the increase in flexural strength of acrylic-modified mortars can be as high as 40%, for prisms made with a polymer/cement ratio of 30% compared to unmodified mortar samples [12]. Finally, besides enhancing strength, polymer modifications can significantly improve mortars’ toughness [12]. In our case, this would be particularly useful to allow *bucatini* and *maccheroni* to survive mechanical mixing.

• Poly(ethylene glycol) (PEG, Sigma Aldrich, Italy): Glycol compounds, particularly the chain molecules of glycol, are known to possess surface-tension reducing properties and, thus, reduce the shrinkage of concrete with a minimal effect on strength [13]. This feature is particularly important as the amount of aggregates in the mixes to be extruded, when present, is very low.
• Plasticizer (Dynamon SP1, Mapei, Italy): This is an admixture based on modified acrylic polymer designed for the concrete precast industry. Concrete prepared with Dynamon SP1 has a high level of workability (consistency class S4 or S5) and is easy to handle when fresh [14].
• Hydroxypropyl methylcellulose (HPMC, Sigma Aldrich, Italy): This was added as a water-retaining agent in some formulations and as a thickening- or viscosity-enhancing admixture, to reduce segregation amongst the components, to improve homogeneity, workability and also the hardened product characteristics [15]. It is demonstrated in the literature that it has a strong influence, not only on the rheological behavior of the mortars, but also on the structural breakdown and reconstruction phenomena during the extrusion process [16,17]. HPMC exerts also a retarding action on setting [15].
• Calcium carbonate (AnalytiCals, Carlo Erba, Italy): This was added as a superfine aggregate in the mixtures of *maccheroni* tubes. Cement containing limestone demands less water than cements with pozzolana and fly ash and exhibits higher early strength [18,19]. Calcite has two functions, one as an active participant in the hydration process, leading to the reduction of the porosity and permeability of hardened cement pastes, and the other as a filler [20]. Therefore, calcium carbonate addition to the mixture is expected to increase the stiffness of the cementitious matrix during the extrusion process, avoiding the collapse of the fresh element.

All the liquids (water, Primal, PEG, plasticizer) were mixed together with an overhead stirrer (Janke and Kunkel IKA, RW 20) while the cement was added progressively. At the end, calcium carbonate was added if necessary. For cementitious hollow tube production, many mix designs were tested, as described in Section 3.1.

#### 2.1.2. Coating and Sealing of the Cementitious Hollow Tubes

In order to obtain a waterproof container for the healing agent, the exterior and interior surfaces of the cementitious hollow tubes were coated with different organic and inorganic materials.

The application of the coating is necessary because the acrylic resin extruded with the cement paste cannot guarantee a sufficient protection of the healing agent. In fact, water is a small molecule that can diffuse through the acrylic macromolecules network and, thus, can leave or can enter the tubes with time. For this reason, an additional coating is aimed at creating a homogeneous film in order to protect the healing agent contained within the tubes from moisture and to avoid the water component of the healing agent being prematurely absorbed by the tube shell. The coating material must fulfill some prerequisites, such as possessing an adequate viscosity and originating a film that is brittle at room temperature and resistant in a basic environment.

Based on these features, two materials were chosen as coating agents. At first, sodium silicate (provided by Sigma Aldrich) was applied on the internal and external surfaces by immersion. To achieve a complete covering of both of the surfaces with a minimal reduction of the internal section, the hollow tubes were immersed into the solution and then hung vertically with clips. In this way, any excess of coating at the bottom of the hollow tubes was removed.

After the first coating, the two extremities of the hollow tubes needed to be sealed in order to provide a container for the healing agent. Wax was selected for this goal because of its hydrophobicity. Moreover, wax is characterized by a rapid curing time and a good adhesion to sodium silicate-coated surfaces. The sodium silicate was then pumped into the cavity of the sealed hollow tube by means of a syringe up to complete filling. To enhance the grip between the wax top and the exterior coating, the wax extremities were covered with a cement paste (with a water-to-cement ratio of 0.3).

Finally, a polyester resin cured in the presence of 2% of methyl-ethyl ketone peroxide (provided by Industria Chimica Reggiana S.p.A., Italy) was applied only to the exterior walls as a second protective coating.

To improve the adhesion between the hollow tubes and the fresh cement paste/mortar in which they had to be placed, the *maccheroni* and *bucatini* were rolled in sand before complete curing of the resin occurred (Figure 2c). Currently, the preparation of *bucatini* and of *maccheroni* is labor intensive, but all of the above described operations can be easily automated with existing technology.

### 2.2. Methods of Analysis for System Validation

#### 2.2.1. Preliminary Characterization of the Cementitious Hollow Tubes

The morphological characterization was carried out by means of a field emission-scanning electron microscope (FE-SEM, HITACHI S-4000). FE-SEM observations were performed on the inner and outer surfaces of both the *bucatini* and *maccheroni* tubes to evaluate the features of the cementitious shells obtained with the different mix designs. These observations were used to select the final composition of the cement paste to be used for the *bucatini* and the *maccheroni* hollow tubes. Furthermore, FE-SEM observations were performed to evaluate the homogeneity of the applied coatings.

The ability of the cementitious hollow tubes to survive a real concrete mixing process was evaluated with the aid of a bottle stirrer (Asal model 724, Italy): a 5-L cylindrical container equipped with a diagonal steel bar was used and filled with concrete, prepared using a water-to-cement ratio of 0.5, a cement-to-aggregate ratio of 1:3 and a coarse-to-fine aggregate ratio of 1:1, by weight. The maximum particle size of the coarse aggregate was 1 cm. Ten hollow tubes (*maccherone* shape) having a length of 5–6 cm were added to the conglomerate. Subsequently, the fresh mass was mixed for 10 minutes by a rotational movement in the same way as happens in a concrete mixer, and then, the number of cementitious hollow tubes surviving to the mix process was evaluated.

The flexural strength of the hollow tubes, *maccherone* shape, was measured on samples with and without polyester resin plus sand coatings in three-point-bending, using an electromechanical testing system (MTS Insight, with a maximum load capacity of 1 kN and standard length, produced by MTS Systems Corporation, Eden Prairie, MN, USA).

X-ray diffraction analysis (XRD, Philips PW 1710) was performed on a pulverized *maccherone* hollow tube to characterize the cement paste. The analysis was done in the range 5–50° 2θ, with a step size of 0.02° and a time per step of 2 s.

#### 2.2.2. Evaluation of the Properties of the Healing Agent

Preliminary tests were performed to characterize the mechanical properties of sodium silicate and its ability to act as a healing agent for cementitious composites, considering that little information is available in the literature about its behavior as a repairing agent [1,4]. More specifically, these tests were aimed at determining the tensile strength (a crucial parameter governing the fracture behavior of brittle materials) of the hardened sodium silicate and to evaluate its ability to form a stable link with a cement matrix. A three-point-bending testing procedure was adopted to achieve this mechanical characterization, as will be detailed in the following, and the basis for comparison was the flexural behavior of a hardened cement paste, *i.e.*, the key component of the cementitious matrix of concretes or mortars.

Cementitious pastes (CEM I 52.5 R) having a water to cement ratio of 0.4 and containing 1% of plasticizer were produced and cast in Plexiglas^®^ molds 7.5 × 2 × 2 cm^3^ in size. Six samples were obtained in this way. They were cured at room temperature for two days in a humid environment (at 20 ± 2 °C and 90% RH), then were demolded and immersed in water at 20 ± 2 °C for 5 days. Finally, they were kept in air in an indoor environment (at 20 ± 2 °C and 50% ± 10% RH), three of them for 1 week and the remaining three for 3 weeks. After curing, the indirect tensile strength of the hardened cement paste was assessed by testing the intact cement specimens thus obtained in three-point-bending, using an electromechanical testing system (MTS Insight 1 kN). The age of the intact cement specimens at the moment of testing was therefore respectively 14 days or 28 days from casting.

After complete failure in bending, sodium silicate was applied by a brush on the fracture surface generated in the middle of the specimens. The two fragments of the original prism were then kept joined together for 7 days in the vertical position without any other external load, in such a way that the hardening process of the sodium silicate could take place, bridging together the opposite edges of the fracture and restoring the integrity of the specimen. Subsequently, the bending test was performed again on the re-assembled specimen thus obtained. In this way, it was possible to obtain a conservative estimate of the indirect tensile strength of the hardened sodium silicate and to evaluate its effectiveness as a binder: indeed, if the second bending test generates a fracture following the path of the first crack, then it can be deduced that the hardened sodium silicate layer failed, and the corresponding peak load can be used to determine the sodium silicate strength; otherwise, if the fracture is created along a different path (*i.e.*, in the cement matrix), then, necessarily, the peak load turns out to be higher than in the first bending test, and the information that can be inferred is that the load bearing capacity of the re-assembled specimen is higher than the intact specimen.

This testing procedure was used to formulate a judgment on the sodium silicate: depending on its capacity to create a stable link between the crack surfaces and to restore, partially or totally, the original flexural strength of the element, the sodium silicate was judged suitable to be used as a healing agent for concrete applications or not. Two different curing ages of the cement paste (14 days and 28 days) were used to take into account the possible effects of further hydration of unreacted cement particles on the mechanical recovery of the re-assembled specimen.

#### 2.2.3. Evaluation of the Self-Healing Effect on the Cementitious Materials

**Figure 3:**
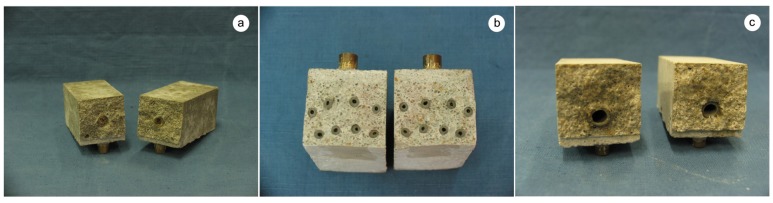
Mortar prisms used for the evaluation of the effectiveness of the proposed self-healing system at the end of 3-point-bending tests: (**a**) sample containing one big hollow tube filled with sand grains (MI series); (**b**) sample with 4 *bucatini* in the lower portion and 4 additional *bucatini* in a central position (BU series); (**c**) sample with only 1 *maccherone* (MS series).

To test the self-healing potential of the proposed systems, mortar specimens measuring 14 × 4 × 4 cm^3^ were produced using a water-to-cement ratio of 0.5 and a cement-to-sand ratio of 1:3 by weight. Two samples without hollow tubes and two samples containing one *maccherone* filled with sand grains instead of sodium silicate (see Figure 3a) were prepared and used as reference specimens for the sake of comparison. Other samples were prepared by adding either *bucatini* or *maccheroni*, previously produced according to the procedure reported in Section 2.1 and filled with sodium silicate. In the first case, during casting, 4 *bucatini* were placed in the lower portion of each prism, and 4 additional *bucatini* were placed in a central position (see Figure 3b); in the second case, the samples were manufactured placing only 1 *maccherone*, approximately at the center of the prism (see Figure 3c). All the samples were cured in a humid environment for 24 h, then demolded and immersed in water for 6 days before testing.

As a whole, 13 specimens were produced: two plain mortar specimens (*i.e.*, Samples 1 and 2 belonging to a control plain mortar series denoted as TQ), two mortar specimens added with a *maccherone* filled with sand (*i.e.*, Samples 1 and 2 belonging to a control series denoted as MI), three mortar specimens added with *bucatini* filled with sodium silicate (Samples 1–3 belonging to a self-healing series denoted as BU) and six mortar specimens added with *maccheroni* filled with sodium silicate (Samples 1–6 belonging to a self-healing series denoted as MS).

**Figure 4:**
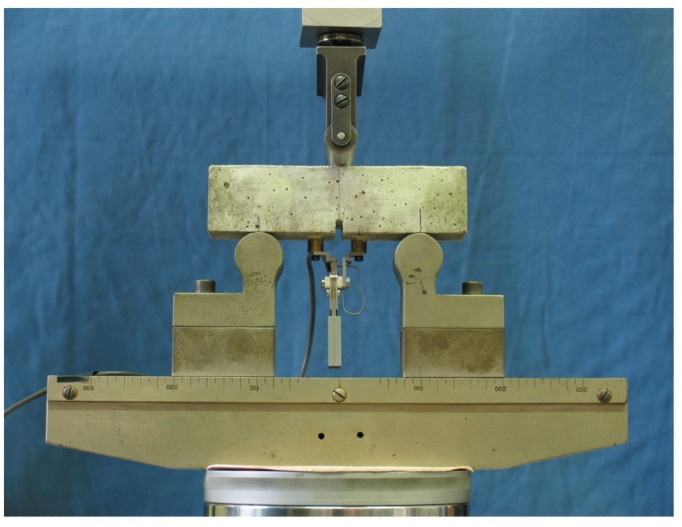
Three-point-bending tests in crack mouth opening displacement (CMOD) control mode.

All the samples were first notched with a U-shaped notch measuring approximately 4 mm in width and 4 mm in height and then subjected to a pre-loading in three-point-bending using a 25-kN closed-loop servo-controlled MTS hydraulic press in crack mouth opening displacement (CMOD) control mode, with a constant rate of 0.002 mm/s and a loading span of 100 mm (see Figure 4). A 25-mm gap clip-on gauge was used to measure the displacement at the gap level during loading.

The specimens were unloaded when the crack opening reached a value between 0.80 and 1.3 mm, regardless of the residual load. The crack was maintained open for 5 minutes at the maximum opening before unloading. The unloading process was conducted with the same CMOD control as for the loading phase, with a rate of 0.002 mm/s. The specimens were removed from the loading frame upon unloading and always stored in a moist environment for 2 days (at 20 ± 2 °C and 90% RH). Then, they were kept in an indoor environment at room conditions (at 20 ± 2 °C and 50% ± 10% RH) for a variable period before the repetition of the test, according to the following:

- -some samples were stored for 10 days (control specimens TQ01, TQ02, MI01 and MI02, self-healing prisms with *bucatini* tubes BU01, BU02 and BU03, self-healing prisms with *maccheroni* tubes MS04 and MS05);
- all of the other samples were stored for 28 days (self-healing specimens with *maccheroni* tubes MS01, MS02, MS03 and MS06).

After these periods of time necessary for the self-healing reactions to be established, all the specimens were tested again in three-point-bending to evaluate their possible performance recovery: they were subjected to a re-loading stage with the same CMOD rate as in the pre-loading stage, up to failure.

Large CMOD values were set in this study, because it is focused on those damage states in concrete structures that could sensibly impair their functioning or durability. Based on the indications reported in the European Standard EN 1992-1-1: Eurocode 2: Design of concrete structures—Part 1-1: General rules and rules for buildings, the CMOD value of 400 μm can be regarded as a threshold discriminating between acceptable crack width in the serviceability limit state (cracks smaller than this do not need a specific control in a variety of structures and exposure classes) and unacceptable crack widths (that require crack control). Therefore, we focused on CMOD values equal to or greater than 800 μm to emphasize this aspect, also considering that the extruded macro-tube system could be easily combined with other established self-healing approaches that efficiently deal with smaller crack widths.

In some cases, a second self-healing effect was manifested, since the two fragments resulting from the complete failure reached in the re-loading stage were manually joined together using an elastic tape and, after some time, appeared to form a cohesive assembly. Therefore, they were subjected to a second re-loading stage, and the performance recovery was evaluated again.

The self-healing effect was evaluated after each re-loading stage through some performance recovery indices, by analogy with a common approach reported in the literature [21]. In particular, a load recovery index *LRI_n_* was defined as:

(1) $$LRI_{n}\left(\%\right)=\frac{P_{n}-P_{u}}{P_{p}-P_{u}}\times 100$$

where *n* is the re-loading cycle index (in our case, *n* = 1 or *n* = 2), *P_n_* is the peak load obtained during the re-loading stage, *P_p_* is the peak load reached during the pre-loading stage (*i.e*., during crack creation in the intact material, corresponding to the material maximum strength) and *P_u_* is the residual load obtained at the moment of unloading preceding the re-loading stage.

In addition, a stiffness recovery index *SRI_n_* was calculated as:

(2) $$SRI_{n}\left(\%\right)=\frac{S_{n}}{S_{p}}\times 100$$

where, again, *n* is the re-loading cycle index (*n* = 1 or *n* = 2), *S_n_* is the stiffness of the specimen during the re-loading stage and *S_p_* is the stiffness of the intact specimen (*i.e*., the stiffness recorded in the pre-loading stage, prior to crack creation). In all cases, the stiffness was defined as the slope of the least-square linear fitting curve of the portion of the load *vs*. CMOD data between 7.5% and 75% of the corresponding peak load.

After the pre-loading stage, the lateral sides of the samples were observed with the microscope DM 4000 M LED by Leica to verify the sodium silicate solution leaking along the fracture and to visually confirm, through a proper magnification, that the healing agent was still liquid and able to flow at the moment of the pre-loading.

X-ray tomography was used to achieve a 3D image of the damage induced inside the prisms in order to verify the diffusion of the healing agent from the hollow tube into the sample and the effectiveness of the self-healing mechanism. The analysis was performed with the instrument phoenix v|tome|x 300, provided by General Electric Company (Wunstorf, Germany), with a tension of 300 kV.

## 3. Results and Discussion

### 3.1. Extrusion of the Hollow Tubes with a Bucatini and Maccheroni Shape

The first goal of the research was to produce by extrusion cementitious hollow tubes suitable to be used as containing/releasing devices for the healing agent. For this purpose, the extruded tubes with different diameters should have a surface free from holes and defects, to avoid dispersion of the healing agent and to prevent any undesired early reaction. In order to select the best composition to obtain tubes characterized by the mentioned features, different mix designs were tested as reported in Table 1 and Table 2.

First of all, hollow tubes with the smallest diameter (*bucatino* shape) were produced. In the first two cases reported in Table 1, no tubes were extruded when only cement, water and plasticizer were used, independently of the water-to-cement ratio and of the amount of plasticizer added. The paste was dusty and unable to be extruded. Consequently, the acrylic emulsion was used to improve the workability of the paste, replacing a portion of water, as reported in the literature [9,10]. In fact, the reported water-to-cement ratio takes into account the amount of water plus the water contained in the acrylic emulsion (about 50 wt% of the resin). *Bucatini* extruded with only PEG or plasticizer (Recipes 3–4) displayed several defects. Homogeneous cementitious hollow tubes were finally extruded when the amount of acrylic resin was increased, keeping constant the water-to-cement ratio, and both plasticizer and PEG were added to the mix (Recipes 6–7). In fact, the PEG, which is a viscosity-enhancing agent (VEA), acts as a lubricant between the paste and the extruder in order to achieve a smooth surface for the extruded objects. Its action is increased by the presence of the plasticizer.

The best mix for the extrusion of *bucatini* was used as a base to obtain the hollow tubes with a larger diameter (*maccherone* shape). To achieve a useful mix for this typology of tube, an increase of the amount of acrylic emulsion and the addition of HPMC were essential. The HPMC, which is a VEA also, plays a complementary role as PEG, because it increases the water retention of the paste and lubricates the grains of the solid particles, limiting the water separation during the extrusion process. In this way, the number of tubes that can be extruded from the same paste was maximized. Nevertheless, the *maccheroni* failed during the extrusion process due to their own weight and to the lower thickness of the walls (Table 2, Recipes 3–4). To solve this problem, calcium carbonate was added as a superfine aggregate (Table 2, Recipe 5). In fact, mixes made of grains having a wide particle size distribution reduce the pressure and limit the drainage effect during the extrusion process and the porosity of the paste.

**Table 1:** Composition details for the extrusion of cementitious hollow tubes with a *bucatini* shape (proportions with respect to cement). HPMC, hydroxypropyl methylcellulose.

Type: *bucatino* shape	W/C ratio	Water (wt%)	Primal (wt%)	PEG (wt%)	Plasticizer (wt%)	HPMC (wt%)	CaCO_3_ (wt%)	Observations
*1*	0.25	25	-	-	1.5	-	-	It was not possible to extrude the paste, which had a dusty texture.
*2*	0.3	30	-	-	2	-	-	It was not possible to extrude the paste, which had a dusty texture.
*3*	0.2	15	10	-	1.5	-	-	The paste was extruded, but the achieved small hollow tubes were punctured. Only a few tubes were obtained.
*4*	0.2	15	10	1.5	-	-	-	The paste was extruded, but the achieved small hollow tubes were punctured. Only a few tubes were obtained.
*5*	0.2	15	10	1	0.5	-	-	The paste was plastic. Small hollow tubes were extruded, but small imperfections were present.
*6*	0.2	12.5	15	1	0.5	-	-	The paste was plastic. Small hollow tubes were extruded, but small imperfections were present.
*7*	0.21	12.5	17.5	1	0.5	-	-	The paste was plastic. Small hollow tubes were extruded with no paste waste. No defects were visible.

**Table 2:** Composition details for the extrusion of cementitious hollow tubes with a *maccheroni* shape (proportions with respect to cement).

Type: *maccherone* shape	W/C ratio	Water (wt%)	Primal (wt%)	PEG (wt%)	Plasticizer (wt%)	HPMC (wt%)	CaCO_3_ (wt%)	Observations
*1*	0.21	12.5	17.5	1	0.5	-	-	No hollow tubes were extruded.
*2*	0.22	12.5	20	1	0.5	-	-	The paste was rubbery. The extrusion was not possible, because of the strong adhesion between the paste and the screw.
*3*	0.22	12.5	20	1	1	-	-	The paste was extruded, but only a few hollow tubes were obtained from the same paste, due to its short time of workability. The surface of the tubes was characterized by many visible defects.
*4*	0.22	12.5	20	1	1	1	-	The paste was extruded, but the hollow tubes had imperfections and holes on the surface.
*5*	0.22	12.5	20	1	1	1	10	The paste was well extruded, and the hollow tubes did not show visible defects. Thanks to the long time of the workability of the mix, many hollow tubes were obtained by the same paste.

### 3.2. Validation of the Cementitious Hollow Tubes in Terms of Morphology, Durability and Preliminary Characterizations

The morphology of both of the typologies of cementitious hollow tubes and the influence of the coatings were examined by FESEM. Concerning *bucatini*, the best results in terms of facility of extrusion, compactness and homogeneity were achieved with the formulation *bucatini* No. 7, as reported in Table 1 and discussed previously. FE-SEM observations highlighted that the walls of this tube were characterized by small and sporadic pores, which were not connected. Considering these features, the small cementitious hollow tubes produced with the mix No. 7 were selected as the containing/releasing devices for the healing agent in the subsequent tests.

**Figure 5:**
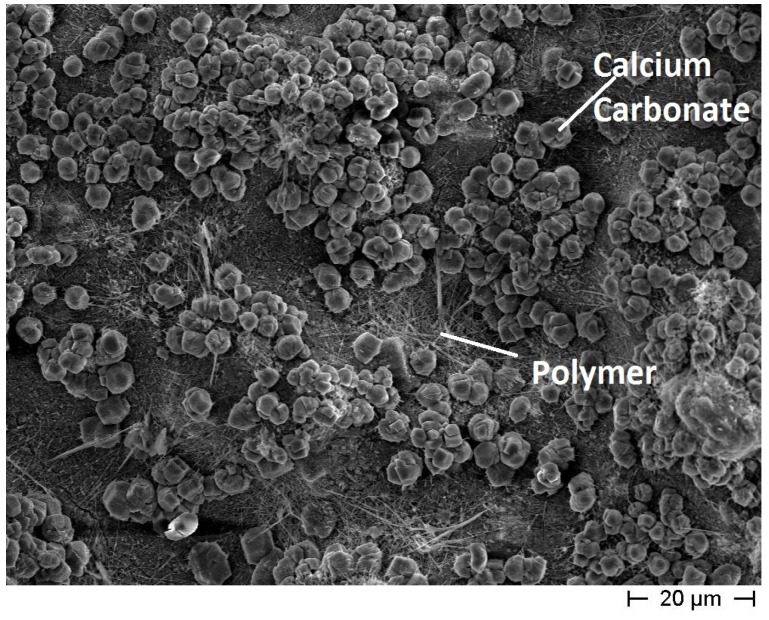
FE-SEM micrograph of the interior surface of the cementitious big hollow tube without coating (recipe for *maccherone* No. 5). The surface is homogeneous and free from defects.

Concerning the *maccherone* shape, the best results were achieved when calcium carbonate was added as an aggregate (recipe for *maccherone* No. 5). As shown in Figure 5, the interior surface of the *maccherone* No. 5 is homogeneous, and the added calcium carbonate appears to be well dispersed in the cementitious matrix. Moreover, the polymer seems to be uniformly distributed on the surface, as well. Actually, it has been reported in the literature that aqueous polymer modifiers have good film-forming capabilities [22]. Finally, in the presence of HPMC in the mix, a major amount of polymer was visible on the surface of the pores [23]. This mix design reported in Recipe 5 was finally adopted for the production of the *maccheroni* to be used in the subsequent tests.

**Figure 6:**
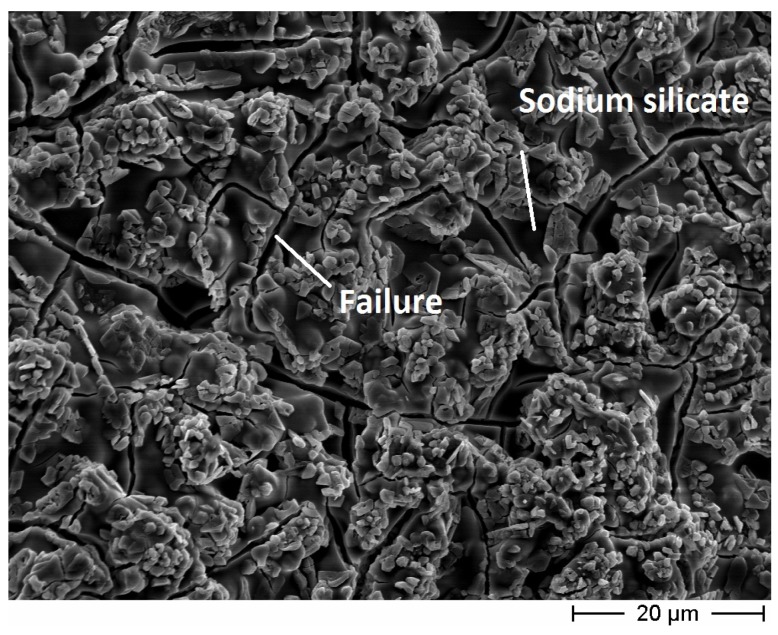
FE-SEM micrograph of the inner wall of the cementitious small hollow tube (recipe for *maccherone* No. 5) coated with sodium silicate. This coating presents some failures.

Considering the coatings, the inner surface of the *maccherone* tube coated with sodium silicate is shown in Figure 6. A homogeneous deposition of the amorphous silica film is visible, even if some cracks are evident. For these reasons, the sodium silicate layer, even if applied onto the two walls (internal and external) of the tubes, was considered insufficient, and a second coating was applied onto the exterior surfaces.

**Figure 7:**
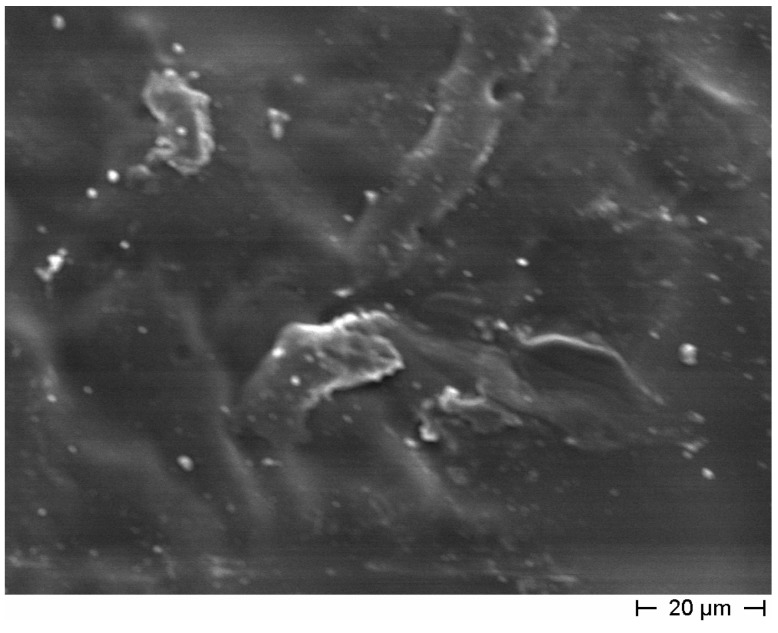
FE-SEM micrograph of the exterior wall of the cementitious small hollow tube (recipe for *maccherone* No. 5) coated with polyester resin. The polymeric layer is homogeneous, free from cracks and completely covers the cement surfaces.

The coating obtained with poly(vinyl) ester resin is visible in Figure 7. In this case, the film is homogeneous and quite thick (about 90 microns), and no cracks were detected. Both coatings (sodium silicate and poly(vinyl) ester resin) were used in the final formulation for *bucatini* and *maccheroni* tubes.

In real conditions, the cementitious hollow tubes should be added to the concrete mix to facilitate their dispersion. For this reason, a preliminary test to evaluate the durability of the hollow tubes during the mixing process was performed, as anticipated in Section 2.1.1. The experimental set-up was built hypothesizing that the rotational movement should be more realistic for the simulation of the mixing process than the one that could be produced using ordinary laboratory equipment, such as a Hobart mixer. The test was performed for 10 minutes and, at the end of the test, all the *maccheroni* were visually and manually controlled, one by one. All the tubes survived the mixing test, namely none of them presented visual damage, loss of integrity or strength reduction.

**Figure 8:**
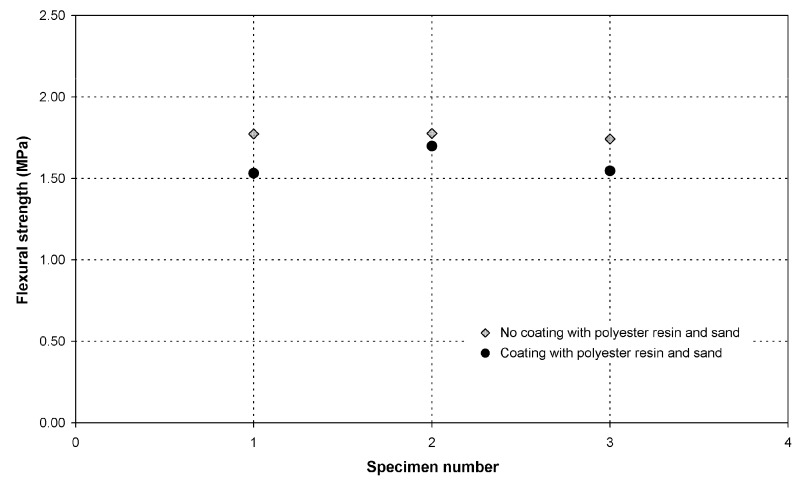
Flexural strength of the hollow tubes, *maccherone* shape, with and without polyester resin/sand coatings (black circles and grey diamond symbols, respectively).

The flexural strength of the hollow tubes with a *maccherone* shape was evaluated assuming that the tube cross-section was a perfectly circular ring whose thickness corresponded to the orifice size of the nozzle. The actual tube thickness was measured after failure using a Vernier caliper with a resolution of 0.05 mm, confirming that the approximation was acceptable. The results are reported in Figure 8. In general, the measured values (ranging between 1.5 and 2 MPa) are characterized by a good reproducibility and are comparable to the flexural strength of normal cement. Moreover, it is evident that the presence of the coating does not significantly influence the mechanical properties of the tubes.

**Figure 9:**
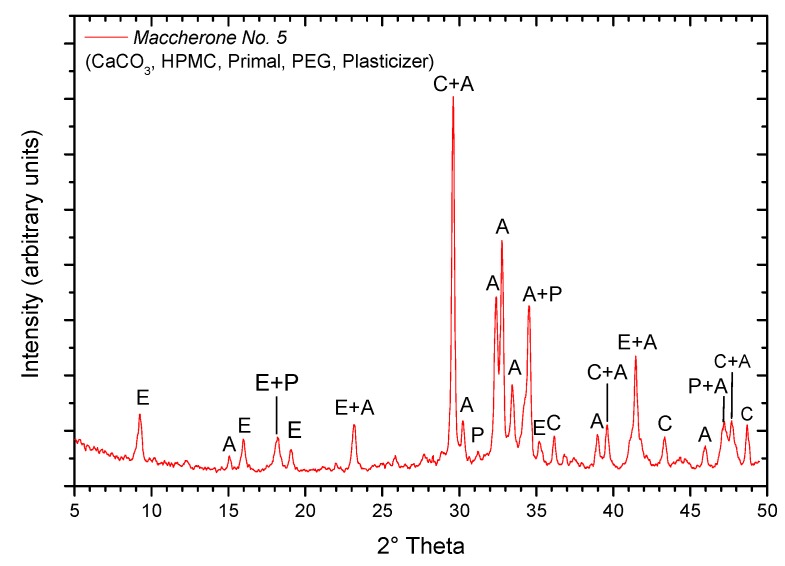
XRD pattern of the extruded *maccherone* hollow tube (Recipe No. 5). C, calcite (CaCO_3_, JCPDS 72-1650); E, ettringite (Ca_6_Al_2_(SO_4_)_3_(OH)_12_.26H_2_O, JCPDS 31-0251); P, portlandite (Ca(OH)_2_, JCPDS 04-0733); A, alite (3CaO.SiO_2_, JCPDS 31-0297).

Considering the number of additives used to extrude the *maccherone* hollow tubes, XRD analysis was performed in order to identify the formed hydrated products. The XRD pattern of the cement paste without any coating is reported in Figure 9: as expected, the main peak was attributable to calcite, and ettringite, portlandite and alite were the main phases. Because C–S–H gel is nearly amorphous, X-ray diffraction can only evidence its presence by the diffuse peak at 0.27–0.31 nm (28.78°–33.16° in 2θ) and the somewhat sharper one at 0.182 nm (50.08° in 2θ) [24]. Finally, the EA/MMA resin being an amorphous polymer, it is not detected by XRD. To conclude, though the complex mix design of the hollow tube paste, the evidenced phases are common in hydrated ordinary Portland cement (OPC) [24]. The same components as in hydrated OPC can be considered as an advantage for being sure that *maccherone* will break when stressed, even if EA/MMA resin addition to the mix should make it less brittle. Considering the possible reaction of hydrated OPC phases with liquid sodium silicate, one should keep in mind the internal coating made of sodium silicate.

### 3.3. Effectiveness of Sodium Silicate as a Healing Agent

**Figure 10:**
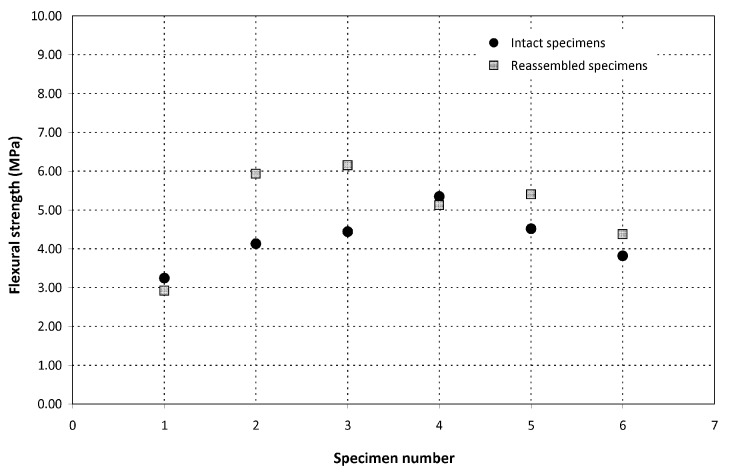
Flexural strength of the intact cement prisms (circles) and of the prisms obtained by re-assembling the residual fragments with sodium silicate (squares). Intact Specimens 1–3 were first tested at the age of 14 days and then tested a second time one week after re-assembling; intact Specimens 4–6 were first tested at the age of 28 days and then tested a second time one week after re-assembling.

The tests performed on the reassembled samples revealed that a complete recovery and sometimes even an enhancement of the flexural strength was obtained with the selected healing agent, as reported in Figure 10. The set of samples reassembled after two weeks of curing and those reassembled after four weeks of curing present a quite similar behavior, thus highlighting that the contribution of autogenous healing due to late hydration of unreacted cement particles can be considered negligible with respect to the healing action generated by the sodium silicate.

### 3.4. Evaluation of the Self-Healing Behavior of Mortars Containing Tubes

**Figure 11:**
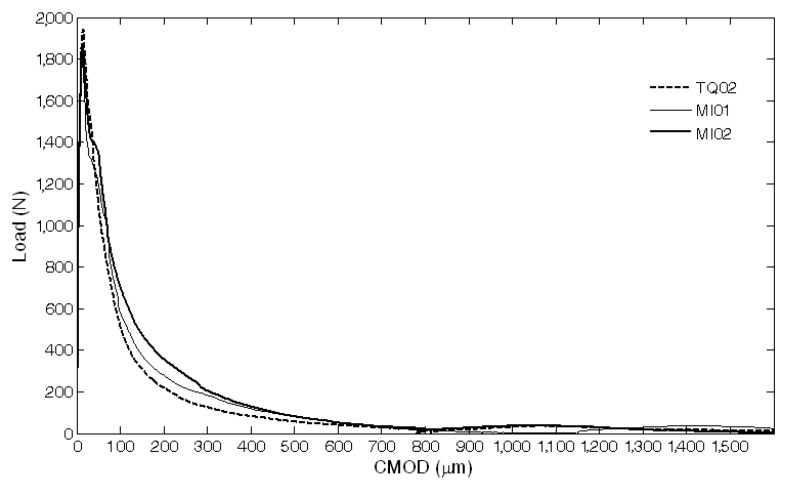
Load *vs*. CMOD curves for control specimens (TQ series and MI series) resulting from pre-loading and re-loading stages.

**Figure 12:**
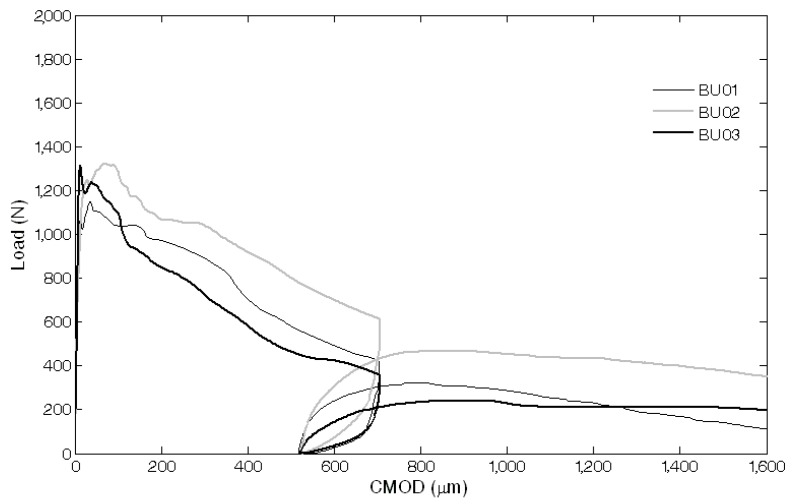
Load *vs*. CMOD curves for self-healing specimens with *bucatini* (BU series) resulting from pre-loading and re-loading stages.

**Figure 13:**
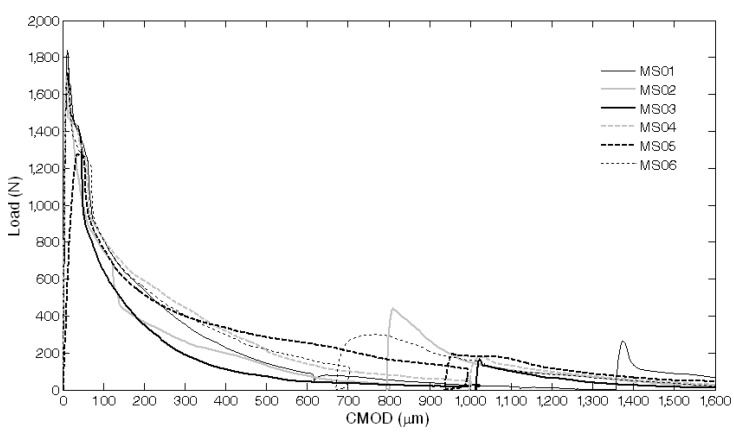
Load *vs*. CMOD curves for self-healing specimens with *maccheroni* (MS series) resulting from pre-loading and re-loading stages.

**Figure 14:**
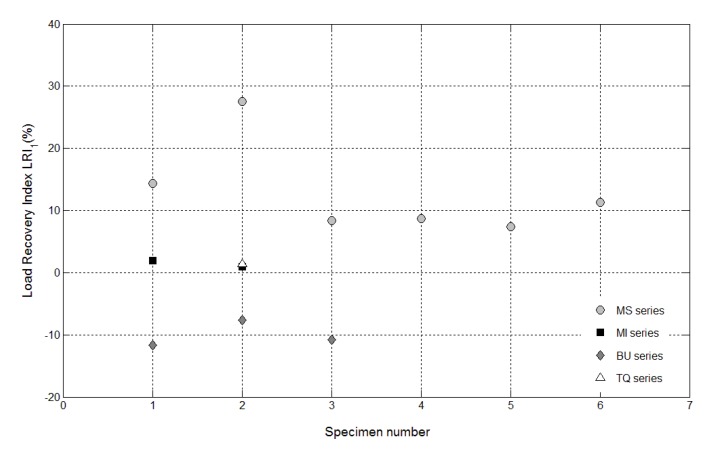
Load recovery indices *LRI_1_* for control specimens (Samples 1 and 2 belonging to the MI series and Sample 2 belonging to the TQ series) and for self-healing specimens (Samples 1–6 belonging to the MS series and Samples 1–3 belonging to the BU series).

**Figure 15:**
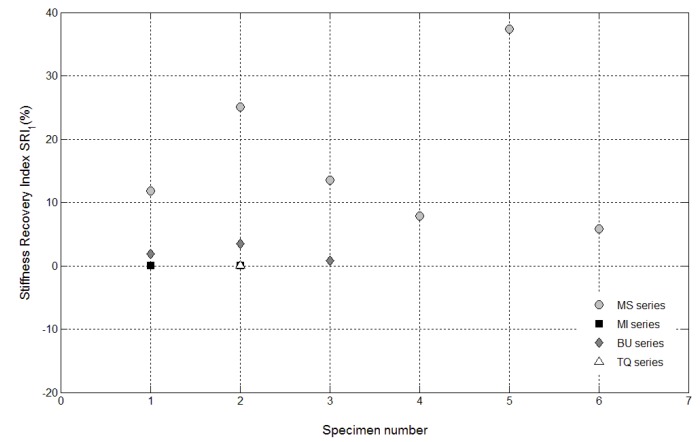
Stiffness recovery indices *SRI_1_* for control specimens (Samples 1 and 2 belonging to the MI series and Sample 2 belonging to the TQ series) and self-healing specimens (Samples 1–6 belonging to the MS series and Samples 1–3 belonging to the BU series).

The results of the mechanical characterization on mortars containing tubes are summarized in Figure 11, Figure 12, Figure 13, Figure 14, Figure 15, Figure 19, Figure 20 and Figure 21. As expected, a very small performance recovery was observed at the end of the re-loading stage for the control specimens (Figure 11, Figure 14 and Figure 15), resulting in a load recovery index ranging approximately from +0.9%–+2% and a stiffness recovery index practically equal to zero. Such a (reduced) self-healing effect has to be ascribed to the well-known phenomenon of further hydration of un-reacted cement particles and is deemed to be insufficient from a mechanical point of view, especially in terms of stiffness recovery. In one case, a control specimen (TQ01) turned out to be broken while being mounted in the loading frame before the re-loading stage, which is an indication of a total absence of self-healing effects.

When the *bucatini* were added, no self-healing effect was observed on the three tested prisms (Figure 12, Figure 14 and Figure 15). This probably happened for the reason that the liquid did not come out of the tubes, presumably because of the capillary resistive force of the cylindrical capsules and of the negative pressure forces caused by the sealed ends that overcame the capillary attractive force of the crack and the gravitational force on the fluid mass [1]. A limited stiffness recovery is balanced by negative values of the load recovery indices, which have to be ascribed to the perturbation of the system caused by the removal of the specimens from the loading frame at the end of each loading stage.

A different behavior was observed when the *maccheroni* were added to the mortars. Indeed successful results were achieved for the first two samples that were tested: 10 days after the pre-loading stage, the load recovery index assumed the value of +8.7% for sample MS04 and of +7.4% for sample MS05 (Figure 14), while the stiffness recovery indices were found to be equal to +7.9% and +37.3%, respectively (Figure 15). Considering the load *vs*. CMOD curves during pre-loading and re-loading stages (Figure 13) and the appearance of the cross-section after rupture (Figure 3), it can be asserted that no tube slipping effects were induced. The good compatibility of the shell material with the surrounding cementitious matrix, the suitable shape and the improvement of the adherence achieved with the application of sand grains to the external surface of the tubes made the cylindrical capsules able to be broken with no slipping after a crack was generated in the cementitious matrix, thus allowing the release of the healing agent without affecting the global mechanical properties of the intact mortar. In fact, the maximum strength, as well as the area subtended by the load *vs*. CMOD curves (related to the samples fracture energy) do not seem to be affected by the incorporation of the big hollow tubes within the prisms.

**Figure 16:**
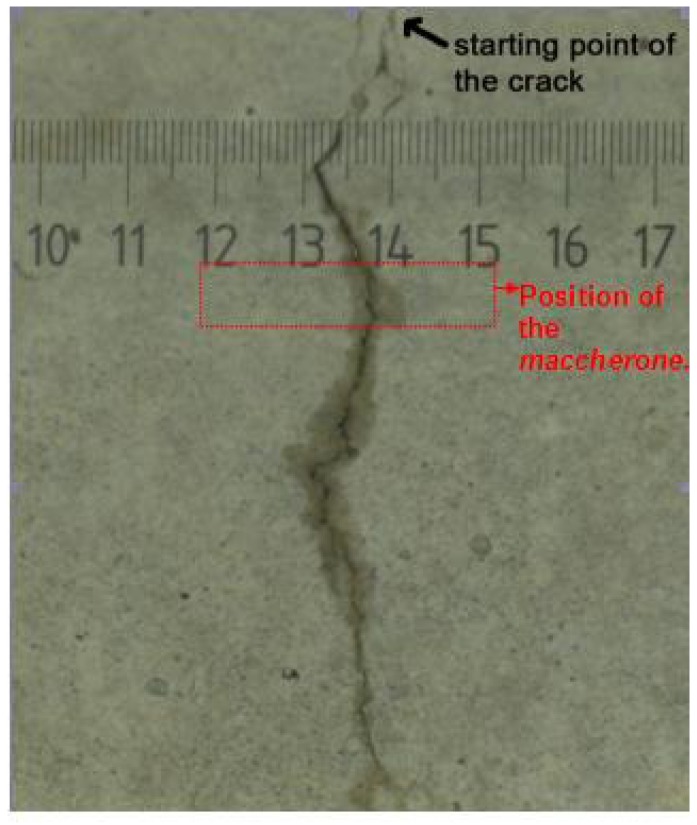
Side view of the specimen with the *maccherone* after the pre-loading stage. The arrow indicates the point of the crack opening; the rectangle indicates approximately the position of the tube inside the matrix. The diffusion of the liquid healing agent through the crack path is clearly visible.

The diffusion of the liquid healing agent from the *maccherone* through the crack path after the pre-loading stage is highlighted in Figure 16. This image, obtained using an optical microscope, evidences that the observed good recovery of the mechanical properties is mainly due to the considerable amount of sodium silicate that is able to flow into the sample after cracking. In the lower portion of the image, it is visible how the capillary forces sucked the liquid where the fracture is thinner.

**Figure 17:**
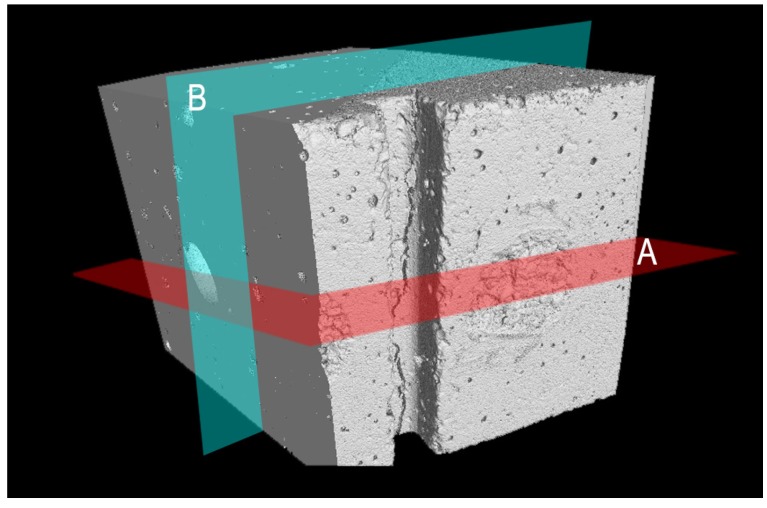
3D visualization of the portion of the cracked prism that contains the *maccherone* tube. The two cross line sections were drawn.

**Figure 18:**
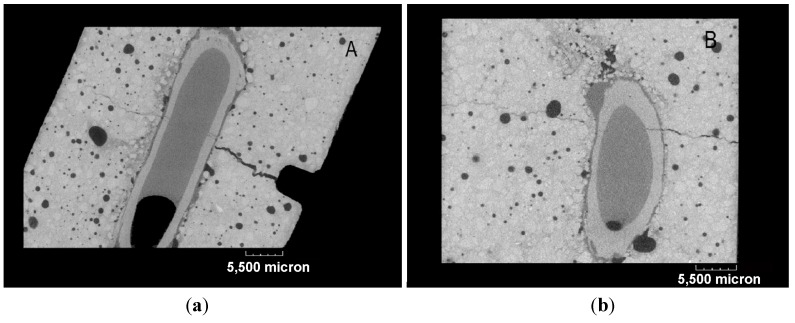
Cross-sections: (**a**) Section Plan A. (**b**) Section Plan B. In both of the sections, cracks crossing the cementitious matrix and the maccherone are clearly visible, as well as the sodium silicate that partially fills the gap between the crack surfaces and the amount of residual healing agent inside the tubular capsule. In the cross-sections, deep-dark colors indicate voids, medium-dark colors indicate liquids and light colors indicate solid areas.

Figure 17 illustrates the 3D rendered dataset of the mortar sample containing the *maccherone* as the container of the healing agent after the prism pre-loading test in three-point-bending. In Figure 17, the U-notch and the fracture are clearly visible. In order to evaluate the amount of healing agent and its diffusion into the sample, cross-sections were realized as drawn in Figure 17. Section Line A cuts the sample in its center, perpendicularly to the crack, as reported in Figure 17 and Figure 18a. Section Line B (Figure 17 and Figure 18b) crosses the *maccherone* that was positioned in the lower portion of the sample. At first, it is possible to visualize the adhesion between the hollow tube and the cement paste, achieved with the application of small aggregates on the surface of the *maccherone.* The sodium silicate is also visible inside the tube and within the cracks: see Figure 18a,b, where deep-dark colors indicate voids, medium-dark colors indicate liquids and light colors indicate solid areas. From Figure 18a, it is clear that only one third of the sodium silicate contained in the tube overflows in the mortar matrix during the crack formation. Despite this, the amount of healing agent is sufficient to fill in the cracks, concentrating at the tip of the crack, as commented before.

A noticeable amount of liquid sodium silicate was observed after breaking the two samples with the big hollow tubes (MS04 and MS05), 10 days after pre-damaging: this result seemed to suggest that healing time could be a significant parameter for a complete reaction of the selected healing agent.

For this reason, it was then decided that the four remaining pre-damaged prisms with *maccheroni* (MS01, MS02, MS03 and MS06) were to be cured for approximately three more weeks, prior to assessment of the performance recovery. Then, one month after pre-loading, the calculated load recovery indices were all positive, ranging from +8.3%–+27.6% (see Figure 14). Similarly, the stiffness recovery indices ranged from +5.8%–+25% (see Figure 15). At first sight, a direct correlation between the curing time of the sodium silicate and the recovery performances and between the crack opening and the recovery index is not noticeable. The variability of these results can be explained considering the natural variability in handmade samples and, in particular, the variable position of the hollow tube within the sample: with respect to the center of the prism section, a difference in height can determine a different opening of the *maccherone* when testing and, consequently, a different release of healing agent.

**Figure 19:**
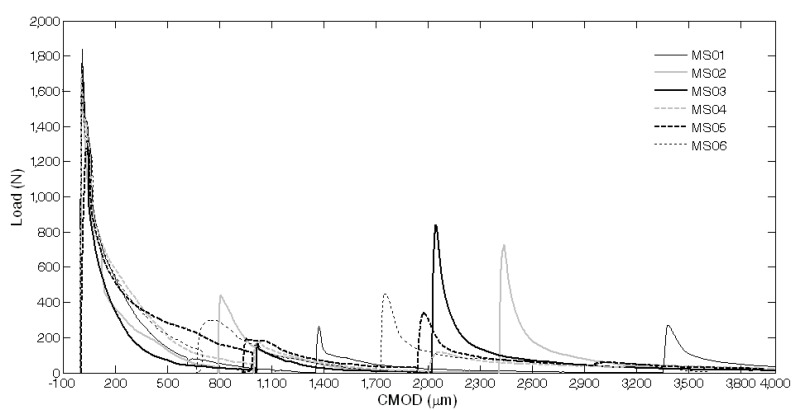
Load *vs*. CMOD curves for specimens with maccheroni (MS series) reassembled after complete failure and re-loaded after the second self-healing process.

**Figure 20:**
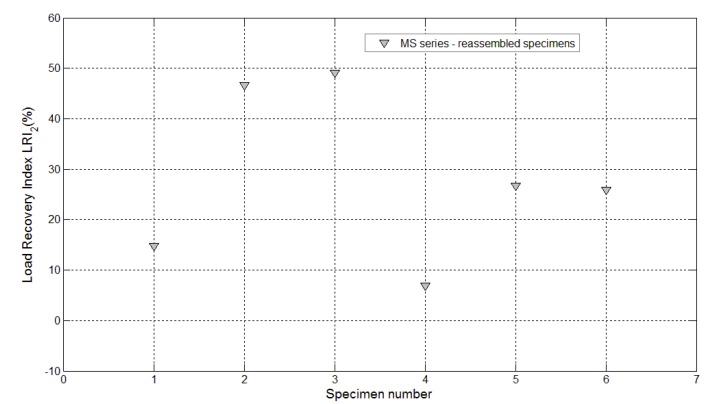
Load recovery indices *LRI_2_* for specimens with maccheroni tubes (Samples 1–6 belonging to the MS series) reassembled after complete failure and re-loaded after the second self-healing process.

**Figure 21:**
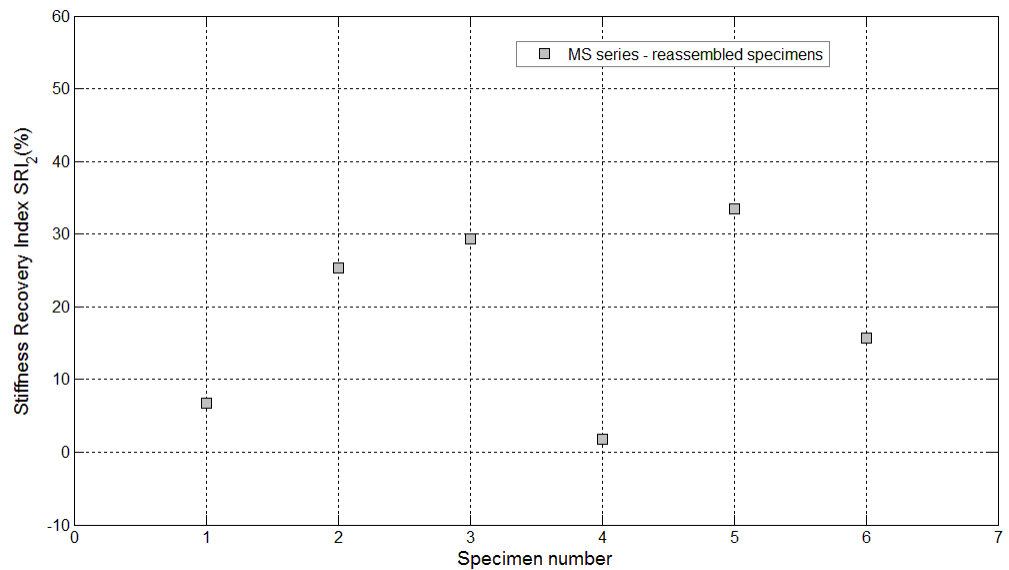
Stiffness recovery indices *SRI_2_* for specimens with maccheroni tubes (Samples 1–6 belonging to the MS series) reassembled after complete failure and re-loaded after the second self-healing process.

Furthermore, in these cases, a certain amount of liquid sodium silicate was observed after breaking the samples. Therefore, finally, all the broken samples were reassembled just after the re-loading stage, by simply joining the two half pieces together with the aid of an elastic tape. Three weeks after the first re-loading stage, the reassembled specimens were subjected to an additional re-loading stage with the same testing conditions and settings as the previous one (see the complete load *vs*. CMOD curves reported in Figure 19), and the performance recovery indices were calculated accordingly (Figure 20 and Figure 21). A surprising further self-healing effect was revealed and is reasonably due to the quite big amount of healing agent, contained in the *maccheroni* tubes, which is made available as soon as the crack opening is increased to such an extent that the fluid could be released once again. Load recovery indices up to nearly 50% and stiffness recovery indices up to 33% were recorded after the second re-loading stage, performed on the reassembled samples. This is a very peculiar aspect of the proposed self-healing system that, in the authors’ opinion, makes it extremely competitive with respect to other solutions already proposed in the literature, also considering the very large crack openings analyzed here in comparison with the smaller values generally investigated in the literature (below 0.22 mm, in [3]).

## 4. Conclusions

In this paper, a novel technique for the production of self-healing concrete has been proposed. Extruded cementitious hollow tubes with different diameters (*bucatini* or *maccheroni*) were produced, and sodium silicate was selected as a proper healing agent.

Several mix designs for the extrusion of *maccheroni* and *bucatini* were investigated. FE-SEM results revealed that the better recipes were characterized by a limited closed porosity in the extruded elements wall. To enhance the durability of both the core and the shell of the hollow tubes, two layers of coating were applied: sodium silicate on the internal and external surfaces of the extruded elements, creating an amorphous film with some cracks, plus a more compact layer of polyester resin, applied only to the external surface. We want to underline here that *maccheroni* were able to keep sodium silicate liquid for several weeks (the healing agent, after pre-damaging and tube rupture, reloading and breaking of the samples one month later, was still liquid, went out of the tubes and was able to repair the samples for a second time). Therefore, the tubes’ waterproofing has been demonstrated. Moreover, the *maccheroni* tubes realized in this way were able to survive a simulated concrete mixing process using a laboratory rotational mixer.

The preliminary results of mechanical characterization tests seem to indicate that sodium silicate was efficiently released by the tubes with a bigger diameter (*maccherone* shape). In this case, a considerable amount of solution was able to diffuse into the mortar cementitious matrix, and a good strength and stiffness recovery was evidenced, even for large cracks of more than 1 mm. A remarkable additional self-healing effect was also observed when further increasing the crack opening after the first self-healing process had taken place. The strength and stiffness recovery after the second self-healing phenomena was even more impressive, confirming the good fluid storage capability of the proposed big cementitious hollow tubes and their potential effectiveness in repairing also multiple/repeated cracks. On the contrary, the core solution contained in the tubes with a lower diameter (*bucatino* shape) was not released, because of the capillary resistive force of the cylindrical capsules and of the negative pressure forces caused by the sealed ends.

Based on these experimental results, it is possible to consider the encapsulation technique presented in this paper as a promising technique. Certainly, further research is needed to corroborate the results from a statistical point of view and also to better understand the behavior and the durability of the healing agent, with regards to its possible solubility in water and potential alkali silica reaction (ASR), and, finally, to evaluate the existing correlation between the viscosity of the agent and the dimension of the tubes and the cracks.
